# Diversity, bioactivities, and metabolomics of culturable endophytic fungi from the medicinal plant *Ardisia crenata* Sims

**DOI:** 10.3389/fmicb.2026.1954910

**Published:** 2026-09-16

**Authors:** Chang Liu, Demei Yang, Jiangli Luo, Sixuan Zhou, Xiongwei Liu, Tingting Feng, Ying Zhou

**Affiliations:** 1 School of Pharmacy, Guizhou University of Traditional Chinese Medicine, Guiyang, Guizhou, China; 2 Guizhou Key Laboratory of Modern Traditional Chinese Medicine Creation, Guiyang, Guizhou, China; 3 Institute of Animal Husbandry and Veterinary Sciences of Guizhou Province, Guiyang, Guizhou, China

**Keywords:** *Ardisia crenata* Sims, bioactive metabolites, endophytic fungi, fungal diversity, metabolomics

## Abstract

**Introduction:**

*Ardisia crenata* Sims is an important medicinal plant in southern China, yet its industrial utilization is severely limited by unsustainable harvesting of wild resources, inconsistent active ingredient quality, and inefficiencies in conventional extraction processes. Endophytic fungi, which can produce bioactive metabolites identical or analogous to those of the host, offer a promising alternative for sustainable biomanufacturing.

**Methods:**

In this study, a comprehensive screening strategy was developed to isolate and evaluate endophytic fungi from *A. crenata* Sims with potential applications as antioxidants, anti-hyperuricemic agents, and anti-diabetic agents.

**Results and discussion:**

A total of 703 culturable endophytic fungal strains were isolated from the roots, stems, leaves, and fruits of plants collected at seven geographical locations, representing 96 genera across three phyla. *Diaporthe*, *Colletotrichum*, and *Cladosporium* were identified as the dominant genera, and tissue-specific colonization patterns were observed. Notably, 20 strains exhibited low ITS sequence homology, indicating that they may represent novel species. Bioactivity-guided screening revealed that strain AGYR3-6 displayed potent DPPH radical scavenging activity (96.64% at 1.0 mg/mL), comparable to that of ascorbic acid. Strain AGYL2-5 exhibited superior xanthine oxidase inhibitory activity (86.88% at 2.0 mg/mL), suggesting anti-hyperuricemic potential. For anti-diabetic activity, strain ABJR1-3 showed strong α-amylase inhibition (84.63% at 2.0 mg/mL), while strain AGYL2-6 exhibited exceptional α-glucosidase inhibition (97.31% at 2.0 mg/mL), outperforming the positive control acarbose. These findings establish a diverse endophytic fungal resource from *A. crenata* Sims and identify multiple candidate strains with promising bioactivities. The results highlight the potential of utilizing these endophytic fungi as microbial cell factories for the sustainable production of bioactive compounds, thereby circumventing the limitations associated with plant-based extraction and supporting the green biomanufacturing of value-added products.

## 1 Introduction

*Ardisia crenata* Sims, a species of the genus Ardisia in the family Primulaceae, is recognized not only as a traditional medicinal plant but also as a promising crop and ornamental plant in southern China. Its roots are commercially valued for their applications in heat-clearing, detoxification, blood circulation activation, and pain relief, and the plant is particularly renowned in Miao medicine for the treatment of sore throat (Wu et al., 2025). Modern phytochemical analyses have demonstrated that *A. crenata* Sims accumulates a range of bioactive compounds (Liu et al., 2022; Tao et al., 2022), which exhibit antibacterial, anti-inflammatory, antiviral, and antitumor activities (Cao et al., 2025; Zhao et al., 2024). Previous phytochemical studies have shown that the roots of *A. crenata* Sims contain diverse bioactive compounds, including triterpenoid saponins, flavonoids, and coumarins (Liu et al., 2016; Liu et al., 2007; Ma et al., 2015; Song et al., 2021). Among these, triterpenoid saponins are considered the major characteristic constituents of the roots (Liu et al., 2016; Liu et al., 2007). Despite its significant medicinal value and promising development prospects, the industrialization of *A. crenata Sims* faces numerous bottlenecks. The medicinal use of this plant relies heavily on wild resources, leading to supply strain due to overexploitation. Artificial cultivation faces challenges including long growth cycles, regional dependence, and continuous cropping issues. Additionally, active component contents vary significantly across geographical origins and tissues, influenced by genetic, environmental, and plant-microbe factors (Liu et al., 2022). Moreover, conventional plant-based extraction processes suffer from high solvent consumption, complex purification, and elevated production costs, hindering industrial development.

To address these industrial challenges, it is essential to understand the biochemical potential of *A. crenata* Sims and the factors that shape its metabolite profile. One informative approach is to investigate the plant endophytic fungal community, which co-evolves with the host and often reflects or contributes to the host’s metabolic capabilities. Endophytic fungi are known to produce secondary metabolites identical or analogous to those of their host plants (Stierle et al., 1993), making them valuable bioindicators of the plant bioactive potential. For *A. crenata* Sims, the endophytic fungal community can serve as a tool to assess the plant’s intrinsic ability to support diverse and potent metabolic pathways, without requiring direct extraction from plant tissues at scale. Importantly, a rich and bioactive endophytic fungal community suggests that the host plant provides a suitable biochemical environment for the production of high-value metabolites, thereby supporting its candidacy for industrial cultivation.

Endophytic fungi, as an important source of bioactive secondary metabolites, have demonstrated application potential in various fields (Newman and Cragg, 2015). In 1993, Stierle et al. isolated a taxol-producing endophytic fungus, *Taxomyces andreanae*, from *Taxus brevifolia*, providing the first evidence for the feasibility of producing plant-derived bioactive substances through microbial fermentation (Stierle et al., 1993). Subsequently, researchers have successively isolated multiple taxol-producing endophytic fungi from plants such as *Ginkgo biloba* (Li et al., 2014; Wu et al., 2018; Yuan et al., 2014; Wu et al., 2022), with some strains exhibiting significantly higher taxol yields than *T. andreanae*. Beyond taxol, significant progress has also been made in the application of endophytic fungi in areas such as antioxidant and hypoglycemic activities. For example, the endophytic fungus *Nigrospora sphaerica* BRN 01, isolated from *Bauhinia purpurea L*., produced fermentation extracts that not only exhibited significant DPPH radical scavenging activity but also demonstrated α-glucosidase inhibitory activity superior to the positive drug acarbose (Kantari et al., 2023). Endophytic fungi isolated from eight medicinal plants used in Nigerian folk medicine were found to contain phenols, tocopherols, sterols, terpenoids, and fatty acids, suggesting their potential for large-scale production of antioxidants to intervene in oxidative stress-related diseases (Ibrahim et al., 2021). A study on *Chloranthus japonicus*, a medicinal plant distributed in China, Japan, and Korea, similarly revealed abundant endophytic fungal resources, with 332 fungal colonies isolated and identified into at least 30 genera, among which *Colletotrichum*, *Aspergillus*, and *Diaporthe* were dominant, and 13 strains exhibited antimicrobial activity (An et al., 2020). Furthermore, endophytic fungi such as *Colletotrichum* have been confirmed as high-yield sources of antimicrobial and antioxidant metabolites (Yehia, 2023). These studies collectively indicate that endophytic fungal community analysis can serve as a rapid and informative screening tool to evaluate the industrial bioactive potential of candidate crop plants prior to large-scale cultivation investment.

Although the value of endophytic fungi in the development of medicinal plant resources has been widely recognized, research on endophytic fungi associated with *A. crenata* Sims remains in its infancy. Existing studies have primarily employed high-throughput sequencing to preliminarily reveal the community composition and diversity characteristics of endophytic fungi in *A. crenata* Sims, identifying Ascomycota and Basidiomycota as the dominant phyla, with significant differences in fungal community structure observed among different geographical origins and tissue types (Liu et al., 2025). To date, no high-throughput screening studies have been conducted to identify endophytic fungal strains from *A. crenata* Sims with specific functional activities such as antioxidant, anti-hyperuricemic, or anti-diabetic properties, nor have core strains with industrial potential and their metabolic characteristics been elucidated. Therefore, using methods such as microbial isolation and cultivation, activity-guided screening, metabolomics analysis, and fermentation performance evaluation, this study will assess the functional activities of candidate strains in terms of antioxidant, anti-hyperuricemic, and anti-diabetic properties. The findings will provide a theoretical basis for the targeted exploration of endophytic fungal resources from *A. crenata* Sims and for green biomanufacturing, promoting the expansion of research on endophytic fungi from this medicinal plant from resource diversity studies to function-driven applied research.

## 2 Materials and methods

### 2.1 Experimental materials and reagents

Fungal DNA Extraction Kit (D2300, Solarbio, Beijing), 1/2 PDA medium, PDB medium, potato, glucose, endophytic fungal fermentation broth, ascorbic acid (Vc), allopurinol, acarbose, methanol, anhydrous ethanol, ethyl acetate, DPPH powder, ABTS, potassium persulfate, xanthine solution, xanthine oxidase solution, HCl solution, anhydrous sodium carbonate, DNS reagent, α-glucosidase solution, PNPG solution, starch solution, α-amylase solution, phosphate buffer, 75% ethanol, 1% sodium hypochlorite solution, sterile water, and glycerol. Instruments: ultra-high performance liquid chromatography coupled with Fourier transform mass spectrometry (UHPLC-Q-Exactive, Thermo Fisher Scientific, USA); BEH C18 chromatographic column (Waters, USA); SW-CJ-2F dual-sided clean bench (Suzhou Purification Equipment Co., Ltd., China); rotary evaporator (EYELA, Tokyo Rikakikai Co., Ltd., Japan); electric thermostatic incubator (Shanghai Pusatong Instrument Co., Ltd., China); and microplate reader (Molecular Devices, USA).

### 2.2 Plant materials

The roots, stems, leaves, and fruits of *Ardisia crenata* Sims were collected between April and June 2024 from seven different locations (Table 1), including Guiyang (GY), Bijie (BJ), Changshun (CS), and Duyun (DY) in Guizhou; Lingshan in Guangxi (GX); Huaihua in Hunan (HN); and Raoping in Guangdong (GD). All samples were morphologically identified as belonging to the genus Ardisia (family Primulaceae). After collection, the samples were placed in ice boxes and transported to the laboratory, where they were stored at 4 °C. Endophytic fungi were isolated within 24 h.

**Table 1:** General information of plant material.

Collection site	Voucher number	Altitude (m)	Longitude (E)	Latitude (N)
Guiyang city, Guizhou	GZ-GY-2024-01	1206.96	106.765326	26.537553
Bijie city, Guizhou	GZ-BJ-2024-02	1511.00	105.2865	27.2858
Changshun country, Guizhou	GZ-CS-2024-03	1241.28	106.321433	26.119169
Duyun city, Guizhou	GZ-DY-2024-04	938.00	107.5157	26.3059
Lingshan country, Guangxi	GX-LS-2024-05	512.00	108.8118	22.4302
Raoping country, Guangdong	GD-RP-2024-06	1210.00	116.8378	24.0681
HuaiHua city, Hunan	HN-HH-2024-07	1884.00	109.9500	27.5000

### 2.3 Isolation of endophytic fungi

Healthy *A. crenata* Sims plants were selected, and surface impurities were removed by rinsing with running water. A soft brush was used to clean the surface attachments of roots, stems, leaves, and fruits. The tissues were cut into 6–8 cm segments, air-dried naturally, and then transferred to a clean bench. Surface sterilization was performed sequentially (Strobel and Daisy, 2003): soaking in 75% ethanol for 1 min, rinsing with sterile water 3–5 times, treatment with 1% sodium hypochlorite solution under shaking (6 min for roots and stems, 4 min for leaves and fruits), followed by another treatment with 75% ethanol for 30 s, and finally rinsing with sterile water 3–5 times. Residual surface moisture was removed using sterile filter paper. The tissues were cut into 0.5 cm × 0.5 cm pieces using a sterile blade and inoculated onto 1/2 PDA medium plates containing chloramphenicol, with five pieces per plate. The plates were sealed and incubated inverted at 28 °C. Meanwhile, an imprint control was set up: sterilized tissue pieces were lightly pressed onto the surface of the culture medium and then removed, followed by incubation under the same conditions. Sterilization was considered effective only when no fungal growth was observed in the control plates.

### 2.4 Purification and preservation of endophytic fungi

From the second day after inoculation, mycelial growth around the tissue pieces was observed daily. When newly grown mycelium appeared, the actively growing region at the leading edge of the mycelium was picked with an inoculation needle in a clean bench and transferred to fresh PDA medium for expanded culture. When the colony diameter reached 3–5 cm, the purity of the strain was assessed. If the colony morphology was uniform and no contamination was observed, subculture was performed and the morphological characteristics of the colony were recorded. If heterogeneous colony morphology or suspected contamination was observed, the different strains were isolated and purified using the gradient dilution method—mycelial tips from different colony edges were picked separately and subjected to three consecutive rounds of isolation culture until morphologically uniform pure cultures were obtained. The purified strains were inoculated onto PDA slants and cultured at 28 °C for 3–5 days. Mycelial plugs were then transferred to cryovials containing 30% glycerol, with three replicates for each strain. The vials were sealed with parafilm, labeled with strain numbers and dates, placed at 4 °C for 2 h, then transferred to −20 °C overnight, and finally stored long-term at −80 °C.

### 2.5 DNA extraction, PCR amplification, and sequencing of endophytic fungi

Genomic DNA of endophytic fungi was extracted using a Fungal DNA Extraction Kit (D2300, Solarbio). PCR amplification was performed with primers ITS1 (5′-TCCGTAGGTGAACCTGCGG-3′) and ITS4 (5′-TCCTCCGCTTATTGATATGC-3′). The PCR reaction system and amplification conditions were set according to the kit instructions. The amplified products were detected by 1% agarose gel electrophoresis and subsequently sent to Sangon Biotech (Shanghai) Co., Ltd. for bidirectional sequencing. Raw sequences were subjected to quality assessment and assembly using DNAStar software. The obtained ITS sequences were analyzed by BLASTn against the NCBI GenBank database, following the BLAST algorithm (Altschul et al., 1990) as implemented in the BLAST+ suite (Camacho et al., 2009). The taxonomic classification of endophytic fungi was preliminarily determined using a sequence similarity threshold of 98%.

### 2.6 Diversity analysis of endophytic fungi from different tissues of *A. crenata* Sims collected from different locations

Based on diversity evaluation indices (Arnold and Lutzoni, 2007), the distribution diversity of endophytic fungi from *Ardisia crenata* Sims was analyzed using isolation rate (IR), isolation frequency (IF), Shannon-Wiener diversity index (H′), Simpson diversity index (D), Sorenson’s similarity coefficient (Cs), and Pielou’s evenness index (J). The Shannon-Wiener index and Simpson index were used to measure species diversity, which is influenced by species richness and community evenness. Under the same species richness, greater evenness among species in the community results in higher H′ and D values, indicating greater species diversity in the sample. Species diversity within a specific habitat is commonly assessed using H′ and D. Generally, higher H′ values and D values closer to 1 suggest greater genetic variation and stronger adaptability to microenvironmental changes. In addition, the Pielou’s evenness index (J) reflects the uniformity of species distribution.

#### 2.6.1 Analysis of isolation frequency and relative isolation rate

Isolation frequency serves as a key parameter for evaluating the community structure of endophytic fungi, primarily used to quantify species diversity and the distribution characteristics of dominant taxa.

The calculation formula is as follows:

Isolation Frequency = Number of strains of a genus/Total number of strains×100%.

#### 2.6.2 Shannon-wiener diversity index (H′) analysis

The Shannon-Wiener diversity index was calculated using the following formula:


$$H^{\prime }=-\sum_{i=1}^{K}P_{i}\times \ln p_{i}$$

where *Pi* represents the proportion of the number of strains of a particular genus of endophytic fungi to the total number of endophytic fungi, and k represents the total number of endophytic fungal species isolated and identified from a specific plant sample or tissue. This formula comprehensively evaluates community diversity by quantifying the evenness and richness of the distribution of different fungal taxa.

#### 2.6.3 Simpson diversity index (D) analysis

The Simpson diversity index was calculated using the following formula:


$$D=1-\sum_{i=1}^{K}{\left(\frac{N_{i}}{N}\right)}^{2}$$

where *Ni* represents the number of individuals of a specific fungal species within the host plant or tissue, *N* is the total number of individuals across all species, and *k* refers to the total number of fungal species. This index reflects the diversity level of the community by quantifying the probability that randomly sampled individuals belong to different species.

#### 2.6.4 Evenness index analysis

The formula for the evenness index is as follows:


E=HlnN

where *H* is the Shannon-Wiener diversity index and *N* is the total number of fungal species.

The formula for the similarity coefficient is as follows:


CS=2j(a+b)

where *j* is defined as the number of endophytic fungal genera shared between two host plants, and *a* and *b* represent the total number of endophytic fungal genera contained in each of the two host plants, respectively. This coefficient assesses the similarity of fungal communities between different hosts by comparing the ratio of shared genera to the total number of genera.

### 2.7 Determination of endophytic fungal activities

#### 2.7.1 Fermentation and extraction of endophytic fungi

Potatoes (200 g) were peeled, cut into pieces, boiled in 1000 mL of ultrapure water for 20–30 min, and filtered through double-layered gauze. Glucose (20 g) was added to the filtrate, dissolved with stirring, and the volume was adjusted to 1,000 mL with ultrapure water. The resulting potato dextrose broth (PDB) culture medium was dispensed into flasks and sterilized. Endophytic fungal strains stored at −80 °C were activated on PDB solid medium and cultured at 28 °C for 3–7 days. Fungal plugs (1 cm × 1 cm) were inoculated into conical flasks containing 200 mL of PDB, with five plugs per flask, and cultured on a rotary shaker at 28 °C and 120 r/min for 7 days. The fermentation broth was filtered under vacuum to separate mycelia from the broth. The filtrate was concentrated under reduced pressure at 50 °C to 120–150 mL, followed by extraction with an equal volume of ethyl acetate 3–5 times. The organic phases were combined and concentrated to dryness under reduced pressure at 50 °C. The residue was dissolved in methanol and then dried in an oven at 37 °C to obtain the crude extract powder (Strobel and Daisy, 2003).

#### 2.7.2 Determination of DPPH radical scavenging activity of endophytic fungi

DPPH powder (2 mg) was accurately weighed and dissolved in 50 mL of anhydrous ethanol to prepare a 0.04 mg/mL DPPH-ethanol solution, which was stored at 4 °C for later use. Different concentrations of test samples were placed in EP tubes, and an equal volume of DPPH-ethanol solution was added to each tube (Munteanu and Apetrei, 2021). After thorough mixing, the mixtures were allowed to react at room temperature in the dark for 30 min. Then, 0.2 mL of the reaction mixture was transferred to a 96-well microplate, and the absorbance was measured at 517 nm. Three parallel replicates were set for each experiment, and ascorbic acid (Vc) was used as a positive control. The scavenging rate was calculated using the following formula: Scavenging rate ((%) = [1 − (A1 − A2) / A0] × 100), where A0 is the absorbance of the mixture of equal volumes of anhydrous ethanol and DPPH-ethanol solution; A1 is the absorbance of the mixture of equal volumes of DPPH-ethanol solution and gradient sample solution; A2 is the absorbance of the mixture of equal volumes of anhydrous ethanol and gradient sample solution.

#### 2.7.3 Determination of ABTS^+^ radical scavenging activity of endophytic fungi

The 5 mL of ABTS^+^ solution and 88 μL of 2.6 mmol/L K₂S₂O₈ solution were accurately measured and mixed in a 50 mL brown volumetric flask. The mixture was allowed to stand at room temperature in the dark for 12 h to obtain the ABTS^+^ stock solution. Before use, the stock solution was diluted with ultrapure water to an absorbance of 0.70 ± 0.02 at 734 nm. Endophytic fungal extracts and the positive control ascorbic acid (Vc) were dissolved in methanol to prepare solutions at concentrations of 0.2, 0.4, 0.6, 0.8, and 1.0 mg/mL. These solutions were prepared immediately before use and stored at 4 °C in the dark (Re et al., 1999).

For each concentration, 200 μL of the endophytic fungal sample solution was mixed with 200 μL of ABTS^+^ working solution in a centrifuge tube and vortexed thoroughly. Separately, 200 μL of the sample solution was mixed with 200 μL of methanol and vortexed thoroughly (control group). A blank group was prepared by mixing 200 μL of ABTS^+^ working solution with 200 μL of methanol. All samples were incubated at room temperature in the dark for 180 min. After incubation, 200 μL of the reaction mixture was transferred to a 96-well plate, and the absorbance was measured at 734 nm. Ascorbic acid (Vc) at the same concentration gradients (0.2, 0.4, 0.6, 0.8, and 1.0 mg/mL) was used as a positive control, following the same procedure. Each concentration gradient was tested in triplicate. The scavenging rate was calculated using the following formula: Scavenging rate = [1 − (A1 − A2) / A0] × 100%, where A1 is the absorbance of the test group: 200 μL sample solution + 200 μL ABTS^+^ working solution; A2 is the absorbance of the control group: 200 μL sample solution + 200 μL methanol; A0 is the absorbance of the blank group: 200 μL methanol + 200 μL ABTS^+^ working solution.

#### 2.7.4 Determination of uric acid-lowering activity of endophytic fungi

Allopurinol was used as a positive control. Four reaction groups were established: the enzyme-added and sample-added reaction group (A), the enzyme-free and sample-added control group (B), the enzyme-added and sample-free blank reaction group (C), and the enzyme-free and sample-free blank control group (D). In a 1.5 mL centrifuge tube, 0.60 mL of phosphate buffer (0.10 mol/L, pH 8.50), 0.20 mL of xanthine solution (2.00 mmol/L), and 0.10 mL of sample solution at different concentrations were added and mixed thoroughly by vortexing. Then, 0.20 mL of xanthine oxidase solution (0.10 U/mL) was added, and the mixture was incubated in a water bath at 37 °C for 30 min. The reaction was terminated by adding 0.20 mL of HCl solution (1.00 mol/L). Each concentration gradient was tested in triplicate. The absorbance was measured at 290 nm, and the uric acid-lowering activity of each endophytic fungus was calculated (Kong et al., 2000).

#### 2.7.5 Determination of hypoglycemic activity of endophytic fungi

Acarbose was used as a positive control to analyze the inhibitory effect of different concentrations of endophytic fungal samples on α-amylase. Sample concentration gradients were set at 0.4, 0.8, 1.2, 1.6, and 2.0 mg/mL. The DNS assay method was employed with 2.00% starch as the reaction substrate. Four reaction groups were established: the enzyme-added and sample-added reaction group (A), the enzyme-free and sample-added control group (B), the enzyme-added and sample-free blank reaction group (C), and the enzyme-free and sample-free blank control group (D). An aliquot of 50.00 μL of sample solution at different concentrations and 50.00 μL of α-amylase solution (1.00 U/mL) were added to a 2.00 mL centrifuge tube, mixed thoroughly by vortexing, and incubated in a water bath at 37 °C for 10 min. Subsequently, 50.00 μL of 2.00% starch solution was added, and the reaction was continued at 37 °C for 20 min. Finally, 100.00 μL of DNS reagent was added to terminate the reaction. The mixture was heated in a boiling water bath for 5 min, then rapidly cooled in an ice water bath, and finally diluted with 1.00 mL of water. The absorbance was measured at a wavelength of 540 nm, and the α-amylase inhibition rate was calculated (Kim et al., 2011).

Similarly, acarbose was used as a positive control to analyze the inhibitory effect of different concentrations of endophytic fungal samples on α-glucosidase. Sample concentration gradients were set at 0.4, 0.8, 1.2, 1.6, and 2.0 mg/mL. PNPG was used as the reaction substrate. Four reaction groups were established: the enzyme-added and sample-added reaction group (A), the enzyme-free and sample-added control group (B), the enzyme-added and sample-free blank reaction group (C), and the enzyme-free and sample-free blank control group (D). An aliquot of 180.00 μL of sample solution at different concentrations and 180.00 μL of α-glucosidase solution (1.00 U/mL) were added to a 2.00 mL centrifuge tube, mixed thoroughly by vortexing, and incubated in a water bath at 37 °C for 15 min. Subsequently, 270.00 μL of PNPG solution was added, and the reaction was continued at 37 °C for 25 min. Finally, 270.00 μL of sodium carbonate reagent was added to terminate the reaction. The absorbance was measured at a wavelength of 405 nm, and the α-glucosidase inhibition rate was calculated.

### 2.8 Detection of chemical components in endophytic fungal fermentation extracts

#### 2.8.1 Sample preparation, chromatographic, and mass spectrometric conditions

Exactly 200 μL of ethyl acetate extract from the fermentation broth of five fungal strains was transferred into a 1.5 mL centrifuge tube, followed by the addition of 800 μL of extraction solvent (methanol:acetonitrile = 1:1, v/v). The mixture was vortexed for 30 s. After low-temperature ultrasonic extraction at 5 °C and 40 kHz for 30 min, the sample was left to stand at −20 °C for 30 min and then centrifuged at 13,000 g and 4 °C for 15 min. The entire supernatant was collected and evaporated to dryness under nitrogen until the ethyl acetate and organic solvents were completely removed. The residue was reconstituted in 120 μL of reconstitution solution (acetonitrile:water = 1:1, v/v), vortexed for 30 s, and subjected to another round of low-temperature ultrasonic extraction at 5 °C and 40 kHz for 5 min, followed by centrifugation at 13,000 g and 4 °C for 10 min. For chromatographic analysis, an ACQUITY UPLC BEH C18 column (100 mm × 2.1 mm, 1.7 μm) was used, with mobile phase A consisting of 2% acetonitrile in water containing 0.1% formic acid, and mobile phase B consisting of acetonitrile containing 0.1% formic acid. The injection volume was 3 μL, and the column temperature was maintained at 40 °C. For mass spectrometric analysis, electrospray ionization was employed, and signals were acquired in both positive and negative ion modes (Chan et al., 2011). The ion source temperature was 450 °C, the capillary temperature was 320 °C, and the scan range was 70–1,050 m/z. The sheath gas flow rate was 50 arb, and the auxiliary gas flow rate was 13 arb. The collision energies were set to 20, 40, and 60%, and the corresponding ion pairs of metabolites eluted within each time segment were monitored.

#### 2.8.2 Data analysis

The raw data were imported into the metabolomics processing software Progenesis QI v3.0 for baseline filtering, peak detection, integration, retention time correction, and peak alignment, yielding a data matrix containing retention time, mass-to-charge ratio, and peak intensity. Feature peak identification was then performed using the same software by matching MS and MS/MS spectral information against the Majorbio in-house proprietary database of Chinese medicine-specific metabolites, with secondary mass spectral matching scores used for comprehensive metabolite identification. Multivariate analyses, including principal component analysis and heatmap analysis, were conducted using the Majorbio data analysis platform. The identified differential metabolites were submitted to the Kyoto Encyclopedia of Genes and Genomes for metabolic pathway enrichment analysis, and cluster heatmaps were generated.

### 2.9 Data statistics and analysis

All experiments in this study were conducted with three biological replicates, and each replicate was measured in three technical replicates. Results are expressed as mean ± standard deviation (Mean ± SD). Statistical analysis was performed using SPSS 26.0 software, and graphs were generated using GraphPad Prism 8.0 software.

## 3 Results

### 3.1 Molecular biological identification of endophytic fungi from *A. crenata* Sims

A total of 703 endophytic fungal strains were isolated from the roots, stems, leaves, and fruits of *Ardisia crenata* Sims collected from seven different locations. Preliminary identification was conducted based on morphological characteristics according to the Manual of Fungal Identification, including mycelial color and texture, colony morphology, colony margin, and reverse side color. The strains exhibited marked differences in colony phenotypic structure, pigment distribution, and substrate characteristics, reflecting rich morphological diversity.

Through ITS sequence-based molecular identification, the 703 strains were classified into 3 phyla, 7 classes, 27 orders, 64 families, and 96 genera (Supplementary Table S1). Among them, Ascomycota accounted for 640 strains (91.0%), comprising 4 classes, 19 orders, 49 families, and 73 genera; Basidiomycota accounted for 60 strains (8.5%), comprising 2 classes, 7 orders, 14 families, and 22 genera; and Mucoromycota accounted for 3 strains (0.4%), comprising 1 class, 1 order, 1 family, and 1 genus.

At the family level, Diaporthaceae (32.69%) and Glomerellaceae (18.80%) were the dominant families. At the genus level, *Diaporthe* was the most abundant genus, with 186 strains showing the highest sequence similarity to this genus, accounting for 26.46% of the total isolated strains. *Colletotrichum* comprised 107 strains (15.23%), and *Cladosporium* comprised 73 strains (10.38%). Collectively, *Diaporthe*, *Colletotrichum*, and *Cladosporium* accounted for 52.07% of the total strains, representing the dominant endophytic fungal genera in *A. crenata* Sims.

### 3.2 Distribution characteristics of endophytic fungal in *A. crenata* Sims from different locations

In terms of the number of isolates from the seven collection locations, 316 strains were obtained from Guiyang, Guizhou (GY); 101 from Raoping, Guangdong (GD); 66 from Changshun, Guizhou (CS); 64 from Lingshan, Guangxi (GX); 59 from Bijie, Guizhou (BJ); 51 from Huaihua, Hunan (HN); and 46 from Duyun, Guizhou (DY) (Figure 1A). At the genus level, GY exhibited the highest endophytic fungal diversity, followed by CS, while GX showed the lowest. *Diaporthe* was a genus common to all seven locations. Each location had its own dominant and unique genera. *Diaporthe* was dominant in GY, GD, and HN; *Colletotrichum* was dominant in BJ and GX; and *Trichoderma* was dominant in DY (Figure 1B). This distribution pattern is closely related to environmental factors such as local climate and soil conditions.

**Figure 1:**
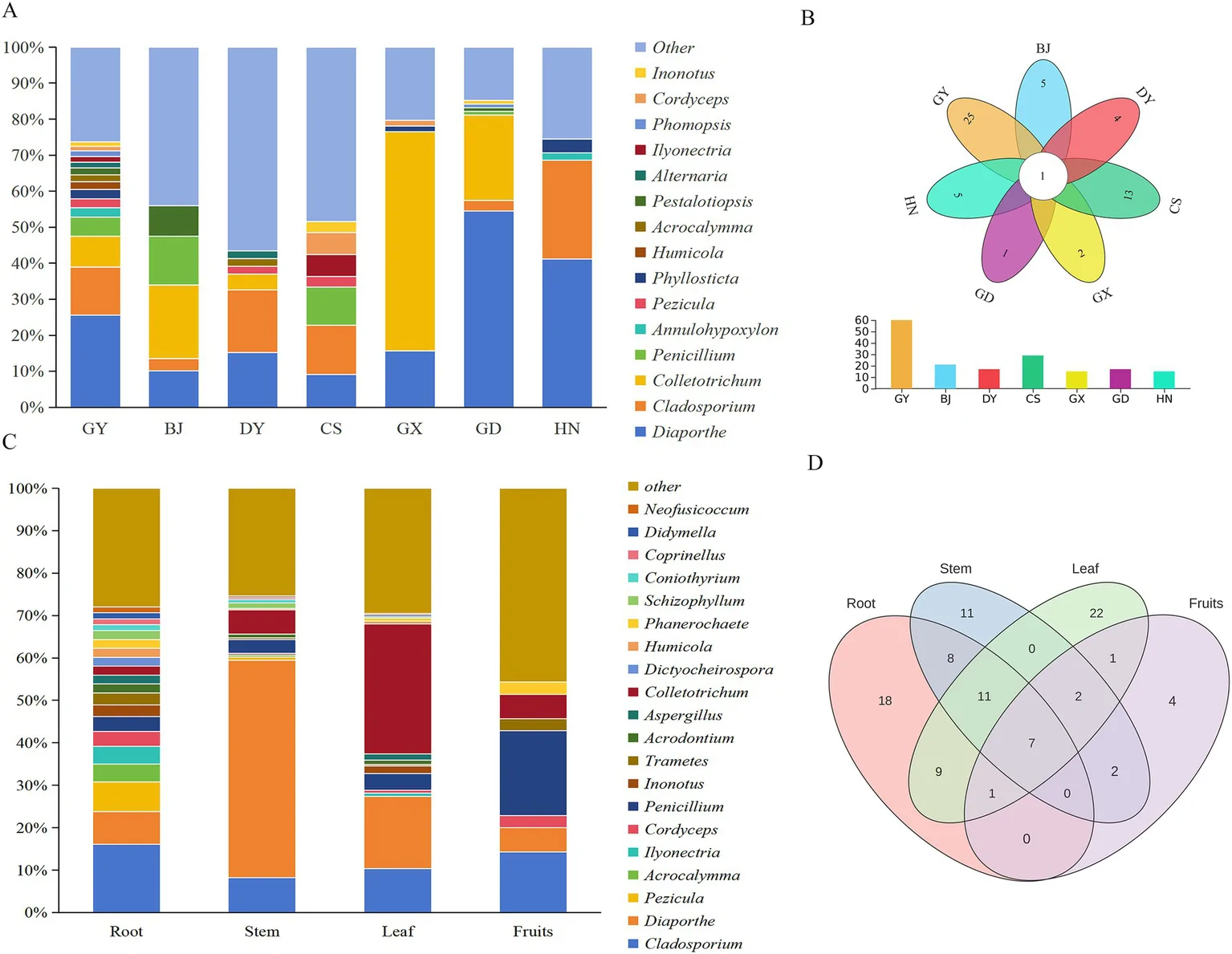
**(A)** Relative abundance of culturable endophytic fungi from *A. crenata* Sims in seven regions at the genus level. **(B)** Venn diagram analysis of culturable endophytic fungi from *A. crenata* Sims in seven regions. **(C)** Relative abundance of culturable endophytic fungi from different tissues of *A. crenata* Sims at the genus level. **(D)** Venn diagram analysis of culturable endophytic fungi from different tissues of *A. crenata* Sim*s.*

In addition, each location harbored specific endemic genera (Supplementary Table S2). GY had 26 endemic genera, including *Aphanocladium*, *Aureobasidium*, *Candolleomyces*, *Ciboria*, *Clonostachys*, *Coniothyrium*, *Curvularia*, *Dactylonectria*, *Dictyocheirospora*, *Ectophoma*, *Geosmithia*, *Guignardia*, *Humicola*, *Inonotus*, *Neometulocladosporiella*, *Ophiosphaerella*, *Pestalotia*, *Plectosphaerella*, *Pleurostoma*, *Polyporus*, *Pseudocercospora*, *Pseudoramichloridium*, *Stemphylium*, *Tetraploa*, *Thelonectria*, and *Umbelopsis*. BJ had five endemic genera: *Botryosphaeria*, *Fusicolla*, *Lentinula*, *Microsphaeropsis*, and *Microsporum*. DY had three endemic genera: *Nemania*, *Parasarocladium*, and *Trichoderma*. CS had 13 endemic genera, including *Acrodontium*, *Botrytis*, *Cutaneotrichosporon*, *Efibula*, *Fomitopsis*, *Lachnum*, *Lophiotrema*, *Moellerodiscus*, *Nigrospora*, *Paraphoma*, *Phlebia*, *Sarocladium*, and *Thanatephorus*. GX had two endemic genera: *Gelatinofungus* and *Resinicium*. GD had one endemic genus: *Pseudogymnoascus*. HN had five endemic genera: *Acremonium*, *Corynespora*, *Leptobacillium*, *Perenniporia*, and *Subuliphorum*.

### 3.3 Distribution characteristics of endophytic fungi in different tissues of *A. crenata* Sims

A total of 36, 20, 46, and 11 fungal genera were isolated from the roots, stems, leaves, and fruits of *Ardisia crenata* Sims, respectively, corresponding to 143, 244, 281, and 35 strains. The dominant genera differed significantly among tissues: *Cladosporium* dominated in roots, with a relative isolation frequency of 16.08%; *Di*aporthe dominated in stems (51.23%); *Coll*etotrichum dominated in leaves (30.60%); and *Penici*llium dominated in fruits (20.00%) (Figure 1C). These results indicated that specific fungal taxa exhibit distinct distribution patterns in different plant tissues, clarifying the core endophytic fungal community structure of *A. crenata* Sims and providing an important basis for understanding plant-microbe interactions.

Fungal community analysis further revealed common and unique taxa among the different tissues. *Cladosporium, Colletotrichum*, *Cordyceps*, *Diaporthe*, *Nigrospora*, *Penicillium*, and *Trametes* were common genera shared among roots, stems, leaves, and fruits (Figure 1D). In addition, each tissue harbored its own specific genera (Supplementary Table S3). The unique genera in roots (18 genera) included *Aphanocladium*, *Ciboria*, *Clonostachys*, *Cutaneotrichosporon*, *Dactylonectria*, *Dictyocheirospora*, *Ectophoma*, *Fusicolla*, *Parasarocladium*, *Perenniporia*, *Pestalotia*, *Phlebiopsis*, *Plectosphaerella*, *Porostereum*, *Pseudoramichloridium*, *Resinicium*, *Thanatephorus*, and *Thelonectria*. The unique genera in stems (12 genera) included *Aureobasidium*, *Corynespora*, *Gelatinofungus*, *Lophiotrema*, *Moellerodiscus*, *Nodulisporium*, *Ophiosphaerella*, *Phomopsis*, *Pleurostoma*, *Stemphylium*, *Tetraploa*, and *Trichoderma*. The unique genera in leaves (23 genera) included *Acremonium*, *Annulohypoxylon*, *Apiospora*, *Botryosphaeria*, *Botrytis*, *Candolleomyces*, *Fomitopsis*, *Geosmithia*, *Guignardia*, *Inonotus*, Lachnum, *Lentinula*, *Leptobacillium*, *Microsphaeropsis*, *Microsporum*, *Nemania*, *Neopestalotiopsis*, *Polyporus*, *Pseudocercospora*, *Pseudogymnoascus*, *Subuliphorum*, *Talaromyces*, and *Umbelopsis*. The unique genera in fruits (4 genera) included *Efibula*, *Paraphoma*, *Phlebia*, and *Sarocladium*.

### 3.4 The α-diversity and PCA analysis of endophytic fungi of *Ardisia crenata* Sims from different sites

#### 3.4.1 α-diversity analysis

Analysis of the α-diversity of endophytic fungal communities in *A. crenata Sims* revealed significant regional differences among samples from different collection sites (Figure 2A). The Shannon-Wiener index values, from highest to lowest, were as follows: CS (5.6787) > GY (2.8745) > BJ (2.4790) > DY (2.2350) > HN (1.6429) > GX (1.4709) > GD (1.3554). The Simpson index values, from highest to lowest, were: CS (0.946) > BJ (0.917) > DY (0.882) = GY (0.882) > HN (0.735) > GX (0.719) > GD (0.603). The Pielou evenness index values, from highest to lowest, were: CS (1.8111) > BJ (0.8941) > DY (0.8714) > HN (0.7135) > GX (0.6694) > GD (0.5455) > GY (0.0625). Overall, CS, GY, and BJ in Guizhou exhibited the highest Shannon-Wiener indices, and their Simpson indices were all close to 1, indicating that *A. crenata* Sims from these three regions possess high genetic variability and environmental adaptation potential.

**Figure 2:**
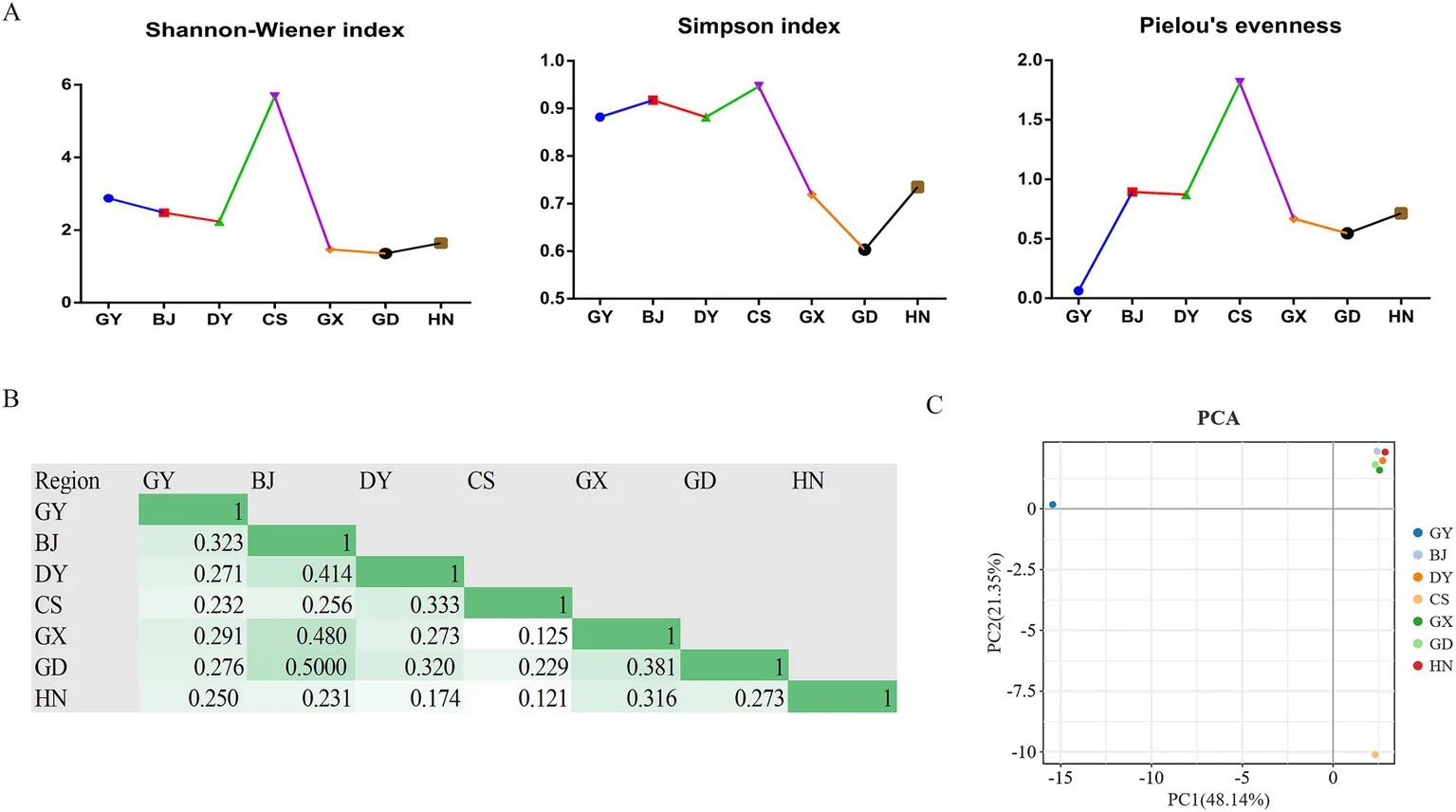
**(A)** Diversity indices of endophytic fungi from *A. crenata* Sims in different regions. **(B)** Similarity coefficients of endophytic fungi from *A. crenata* Sims in different regions. **(C)** PCA analysis of endophytic fungi from *A. crenata* Sims in different regions.

#### 3.4.2 Similarity coefficients of endophytic fungi from different collection sites

Similarity coefficients were used to assess the degree of similarity in the species composition of endophytic fungi in *A. crenata* Sims from different regions (Figure 2B). The similarity coefficients of endophytic fungi from the seven regions ranged from 0.121 to 0.500. The highest similarity coefficient (0.500) was observed between BJ and GD, followed by that between BJ and GX (0.480). The lowest similarity coefficient (0.121) was found between CS and HN. PCA revealed that the distribution distances of regional samples in the principal component space were highly consistent with the clustering results based on similarity coefficients (Figure 2C). Specifically, sample points from BJ were clustered closely with those from DY, GX, GD, and HN. In contrast, sample points from CS and GY were the farthest apart, located at opposite ends of the scatter plot. These findings further confirm that significant geographical differences exist in the community structure of endophytic fungi in *A. crenata* Sims from different regions.

### 3.5 The α diversity and PCA analysis of endophytic fungi in different tissues of *A. crenata* Sims

#### 3.5.1 α diversity analysis

This study analyzed the diversity characteristics of endophytic fungal communities in the roots, stems, leaves, and fruits of *A. crenata* Sims (Figure 3A). The Shannon-Wiener diversity indices for the four tissues, ranked from highest to lowest, were roots (3.8178) > leaves (3.4830) > stems (2.6840) > fruits (2.5053), indicating that the root fungal community had the richest species composition and the highest diversity. This suggests that roots may provide a more heterogeneous microhabitat or a relatively permissive immune environment, thereby accommodating a greater variety of fungal taxa. The Simpson indices were roots (0.985) > fruits (0.954) > leaves (0.949) > stems (0.827). Stems and leaves exhibited high similarity in both diversity indices, possibly reflecting a certain degree of similarity in the composition or ecological functions of their endophytic fungal communities. The evenness indices showed only small differences among tissues, with roots (1.0654) > fruits (1.0448) > leaves (0.9097) > stems (0.8959), indicating that although significant differences exist in fungal species composition among different tissues, the within-community evenness remained relatively high.

**Figure 3:**
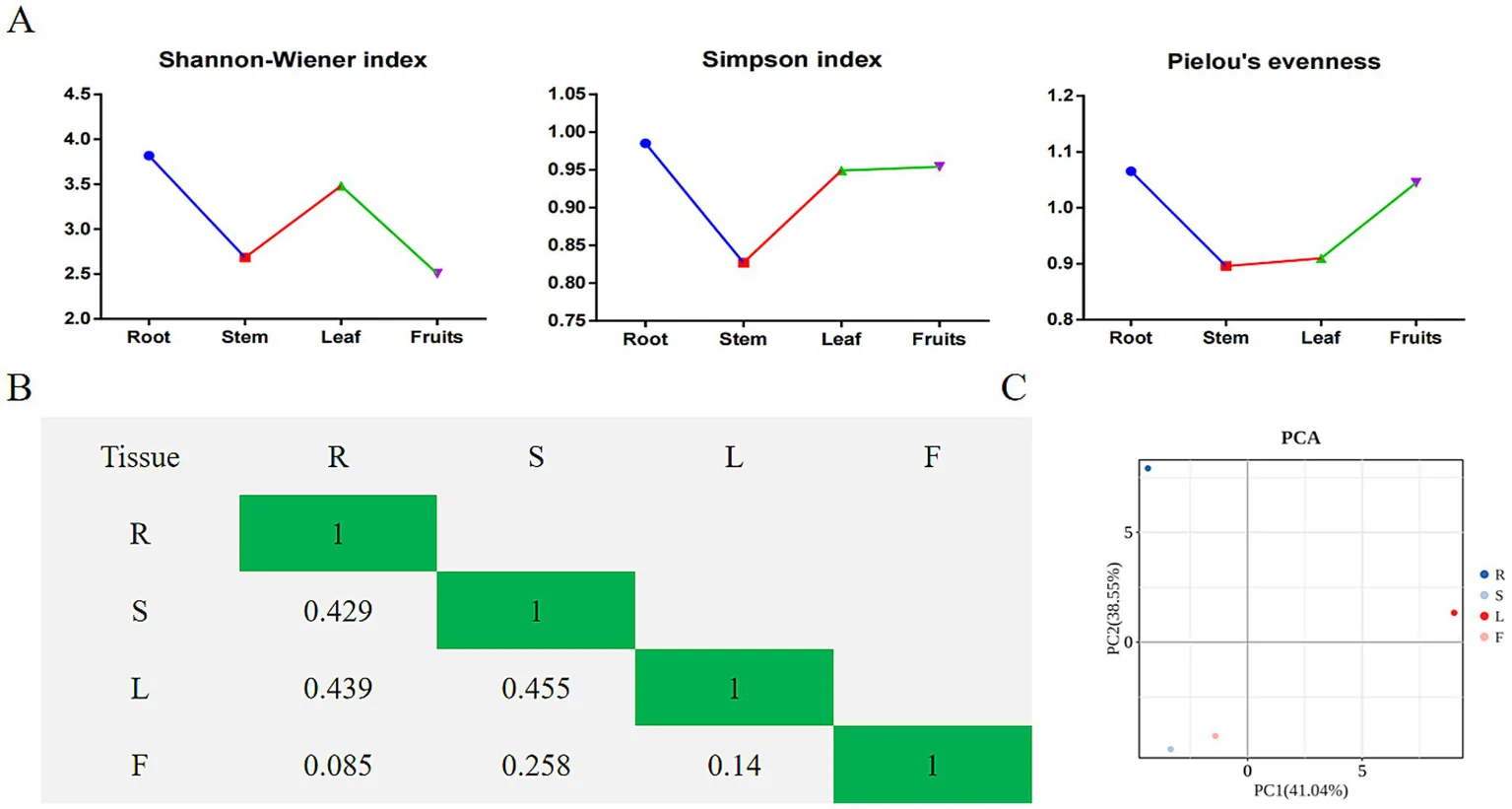
**(A)** Diversity indices of endophytic fungi from different tissues. **(B)** Similarity analysis of endophytic fungi from different tissues. **(C)** PCA analysis of endophytic fungi from different tissues.

#### 3.5.2 Similarity coefficients of endophytic fungi in different tissues of *A. crenata* Sims

Similarity coefficients were used to evaluate the degree of similarity in the species composition of endophytic fungi among different tissues of *A. crenata Sims* (Figure 3B). The similarity coefficients for the four tissues (roots, stems, leaves, and fruits) ranged from 0.085 to 0.455. The highest similarity was observed between stems and leaves (0.455), followed by roots and leaves (0.439). The similarity between roots and stems was 0.429, between leaves and fruits was 0.140, and the lowest similarity was found between roots and fruits (0.085). PCA revealed that samples from different tissues formed clearly separated clusters in the principal component space (Figure 3C). The sample points for stems and fruits were located close to each other, indicating a relatively similar fungal community composition. In contrast, the sample points for roots and leaves were distinctly separated from the other two tissues, revealing their unique fungal community structures.

### 3.6 Homology alignment results

Through homology alignment, a total of 20 strains with homology below 98% were obtained, with values ranging from 88.48 to 97.90% (Supplementary Table S4; Figure 4). Among them, the strain with the lowest homology was AGYL2-1 (*Waltergamsia harwoodiae*), at only 88.48%, while the strains with the highest homology were AGYL2-2 (*Penicillium sumatraense*) and ABJR1-3 (*Fusicolla filiformis*), each reaching 97.90%. The remaining strains, such as AGYR3-7 (*Penicillium brevicompactum*), ACSS1-5 (*Diaporthe jinxiu*), and ADYS3-2 (*Aspergillus aculeatus*), exhibited homology values ranging from 93 to 98%, demonstrating considerable sequence divergence. These results suggest that these strains may represent potential new species or taxa whose taxonomic status remains to be clarified.

**Figure 4:**
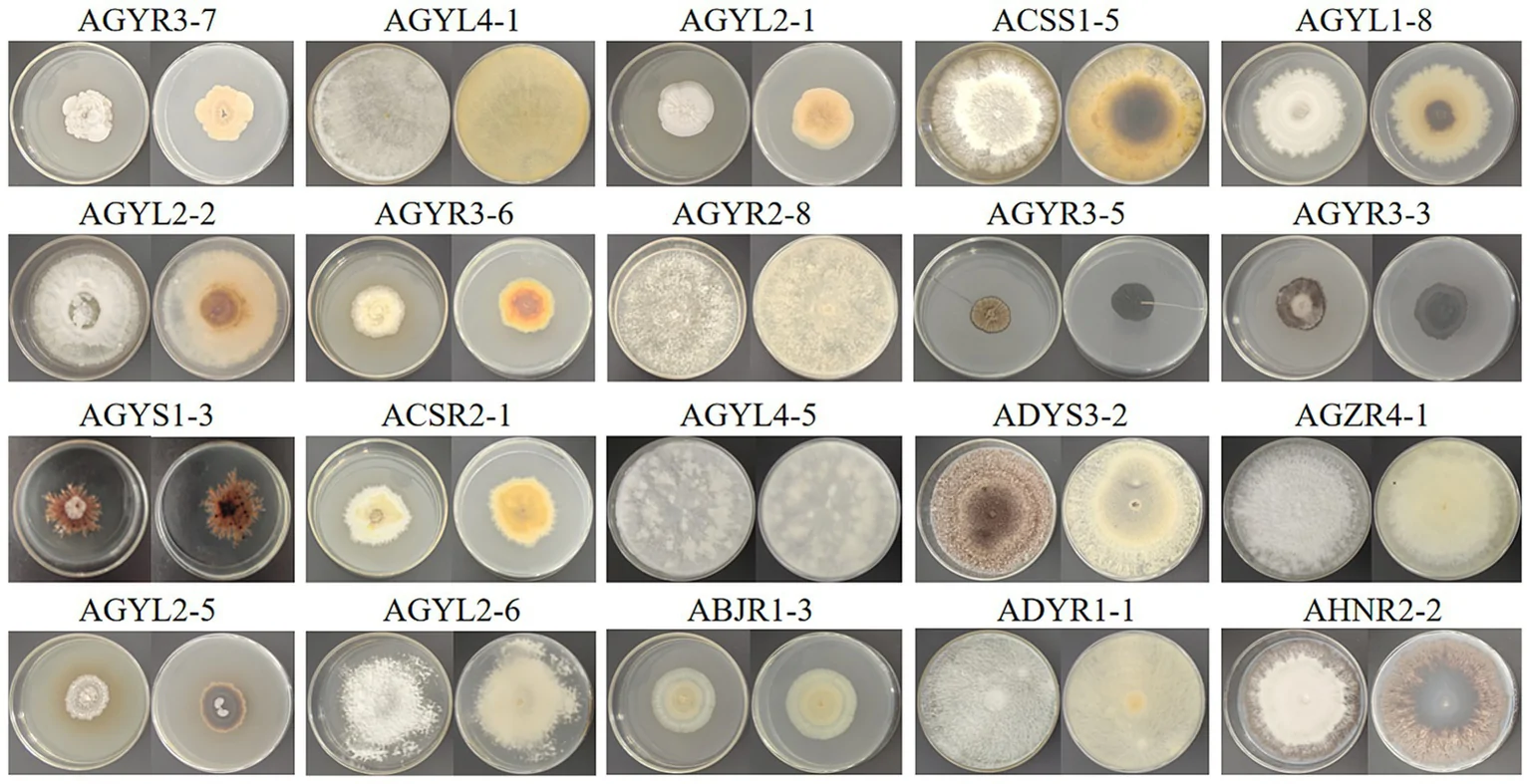
Front and back views of colonies of 20 endophytic fungal strains.

### 3.7 Determination of endophytic fungal activity

#### 3.7.1 DPPH radical scavenging activity assay

Using ascorbic acid as a positive control, the DPPH radical scavenging rates of endophytic fungal samples at different concentrations were determined (Supplementary Table S5). The results are shown in the figure. Within the concentration range of 0.2–1.0 mg/mL, the DPPH radical scavenging rates of all endophytic fungi increased with rising mass concentrations, exhibiting a certain dose-dependent effect. At the highest concentration (1.0 mg/mL), the endophytic fungal strain AGYR3-6 showed the best scavenging activity, reaching 96.64% ± 0.21%, which was comparable to that of the positive control group (Figure 5A). Molecular identification revealed that this strain is closely related to MF948626.1 and may be *Dictyocheirospora rotunda*.

**Figure 5:**
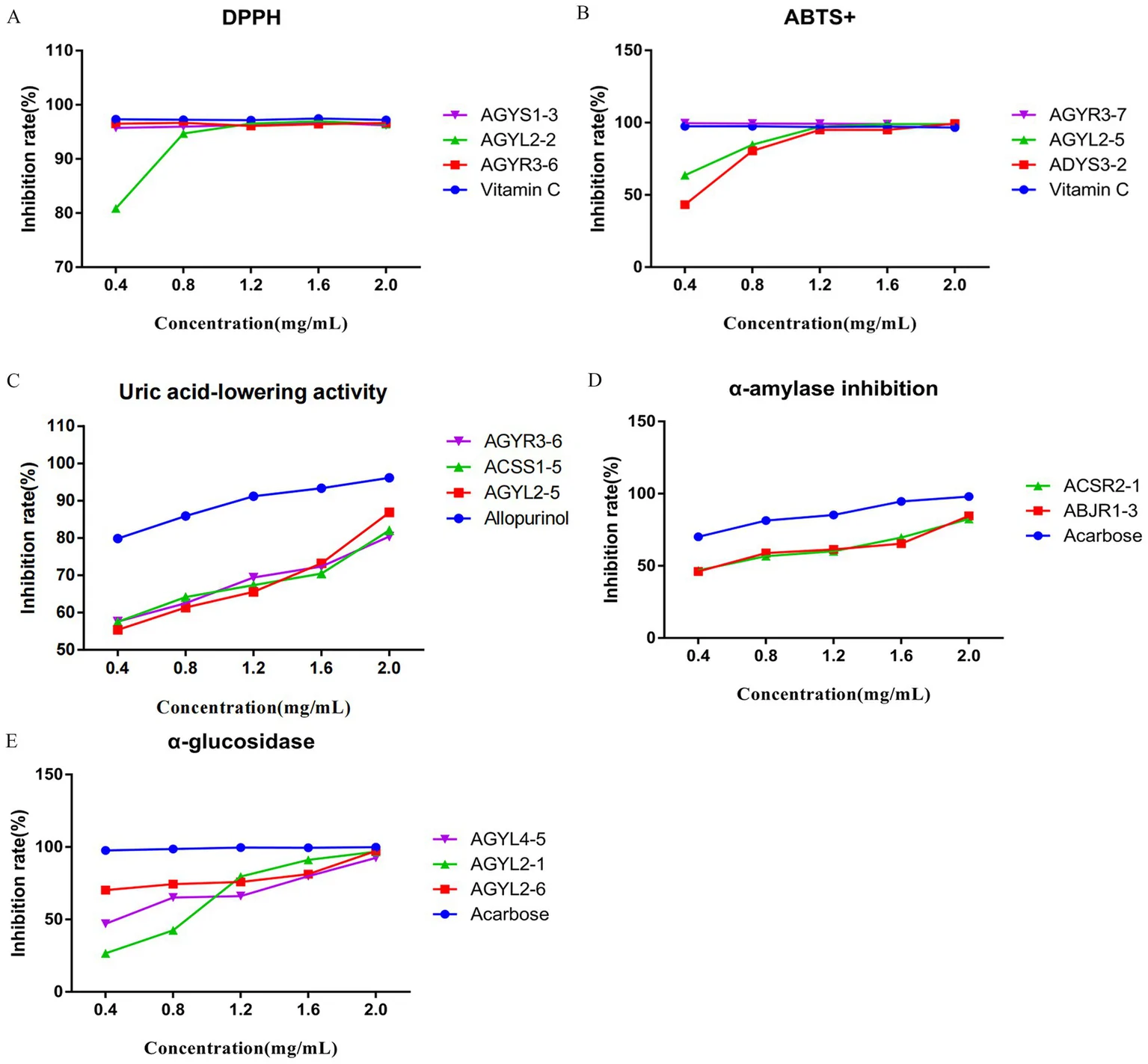
Determination of endophytic fungal activity: **(A)** The DPPH radical scavenging rates of endophytic fungal samples. **(B)** The ABTS^+^ radical scavenging rates of endophytic fungal samples. **(C)** The xanthine oxidase inhibitory rates of endophytic fungal samples. **(D)** The α-amylase inhibitory rates of endophytic fungal samples. **(E)** The α-glucosidase inhibitory rates of endophytic fungal samples.

#### 3.7.2 ABTS^+^ radical scavenging activity assay

Using ascorbic acid as a positive control, the ABTS^+^ radical scavenging rates of endophytic fungal samples at different concentrations were determined (Supplementary Table S6). Within the concentration range of 0.2–1.0 mg/mL, the ABTS^+^ radical scavenging rates of all endophytic fungi increased with rising mass concentrations, exhibiting a dose-dependent effect. When the concentration reached a certain level, the scavenging effects of all strains were comparable to that of the positive control group. At the highest concentration (1.0 mg/mL), the endophytic fungal strain ADYS3-2 exhibited the strongest scavenging activity, reaching 99.39% ± 1.00%, which was comparable to that of the positive control group (Figure 5B). Molecular identification revealed that this strain is closely related to ON965492.1 and may be *Aspergillus aculeatus*.

#### 3.7.3 Determination of uric acid-lowering activity

Using allopurinol as a positive control, the inhibitory rates of endophytic fungal samples at different concentrations on xanthine oxidase (XOD) were determined (Supplementary Table S7). Within the concentration range of 0.40–2.00 mg/mL, the XOD inhibitory rates of the endophytic fungal samples increased with rising sample concentrations. At the highest concentration (2.0 mg/mL), ACSS1-5, AGYR3-6, and AGYL2-5 exhibited XOD inhibitory rates exceeding 80%. Among them, the endophytic fungal strain AGYL2-5 showed the strongest inhibitory effect, with an inhibition rate of 86.88% ± 0.35% (Figure 5C). Molecular identification revealed that this strain is closely related to NR168784.1 and may be *Muyocopron heveae*.

#### 3.7.4 Determination of hypoglycemic activity

##### 3.7.4.1 α-Amylase inhibition assay

Using acarbose as a positive control, the inhibitory rates of endophytic fungal samples at different concentrations on α-amylase were determined (Supplementary Table S7). Within the concentration range of 0.40–2.00 mg/mL, the α-amylase inhibitory rates of the endophytic fungal samples increased with rising sample concentrations. At the highest concentration (2.0 mg/mL), AGYR3-7, AGYL1-8, AGYR3-6, AGYR2-8, AGYR3-5, and ADYR1-1 exhibited α-amylase inhibitory rates exceeding 70%; ACSR2-1 and ABJR1-3 showed inhibitory rates exceeding 80%. Among them, the endophytic fungal strain ABJR1-3 demonstrated the best inhibitory effect, with an inhibition rate of 84.63% ± 0.07% (Figure 5D). Molecular identification revealed that this strain is closely related to OQ128332.1 and may be *Fusicolla filiformis.*

##### 3.7.4.2 α-Glucosidase inhibition assay

Using acarbose as a positive control, the inhibitory rates of endophytic fungal samples at different concentrations on α-glucosidase were determined (Supplementary Table S8). Within the concentration range of 0.40–2.00 mg/mL, the α-glucosidase inhibitory rates of the endophytic fungal samples increased with rising sample concentrations. At the highest concentration (2.0 mg/mL), AGYL2-1, AGYL4-5, and AGYL2-6 exhibited α-glucosidase inhibitory rates exceeding 90%; AGYR3-7, AGYL1-8, AGYL2-2, AGYR3-6, AGYR3-5, AGYS1-3, and AGZR4-1 showed inhibitory rates exceeding 80%; AGYR2-8, ADYS3-2, AGYL2-5, and AHNR2-2 showed inhibitory rates exceeding 70%. Among them, the endophytic fungal strain AGYL2-6 demonstrated the best inhibitory effect, with an inhibition rate of 97.31% ± 0.01% (Figure 5E). Molecular identification revealed that this strain is closely related to OL981961.1 and may be *Mycoleptodiscus terrestris*.

### 3.8 Chemical composition analysis of five strains with high bioactivity

To elucidate the chemical basis of the five highly active endophytic fungal strains AGYR3-6, ADYS3-2, AGYL2-5, ABJR1-3, and AGYL2-6, untargeted metabolomics analysis was performed on the ethyl acetate extracts of their fermentation broths using UPLC-MS/MS. Mass spectrometry data were acquired in both positive and negative ion modes. The total ion current chromatograms exhibited stable baselines, well-resolved peak shapes, and uniform retention time distribution, indicating reliable data quality suitable for subsequent qualitative and comparative analyses (Figure 6). By matching the MS/MS spectra against natural product databases, a total of 1,071 secondary metabolites were identified. Among these, 875 metabolites (81.7%) were detected in positive ion mode, while 196 metabolites (18.3%) were detected in negative ion mode, indicating that positive ion mode yielded substantially more metabolite identifications (Figure 6).

**Figure 6:**
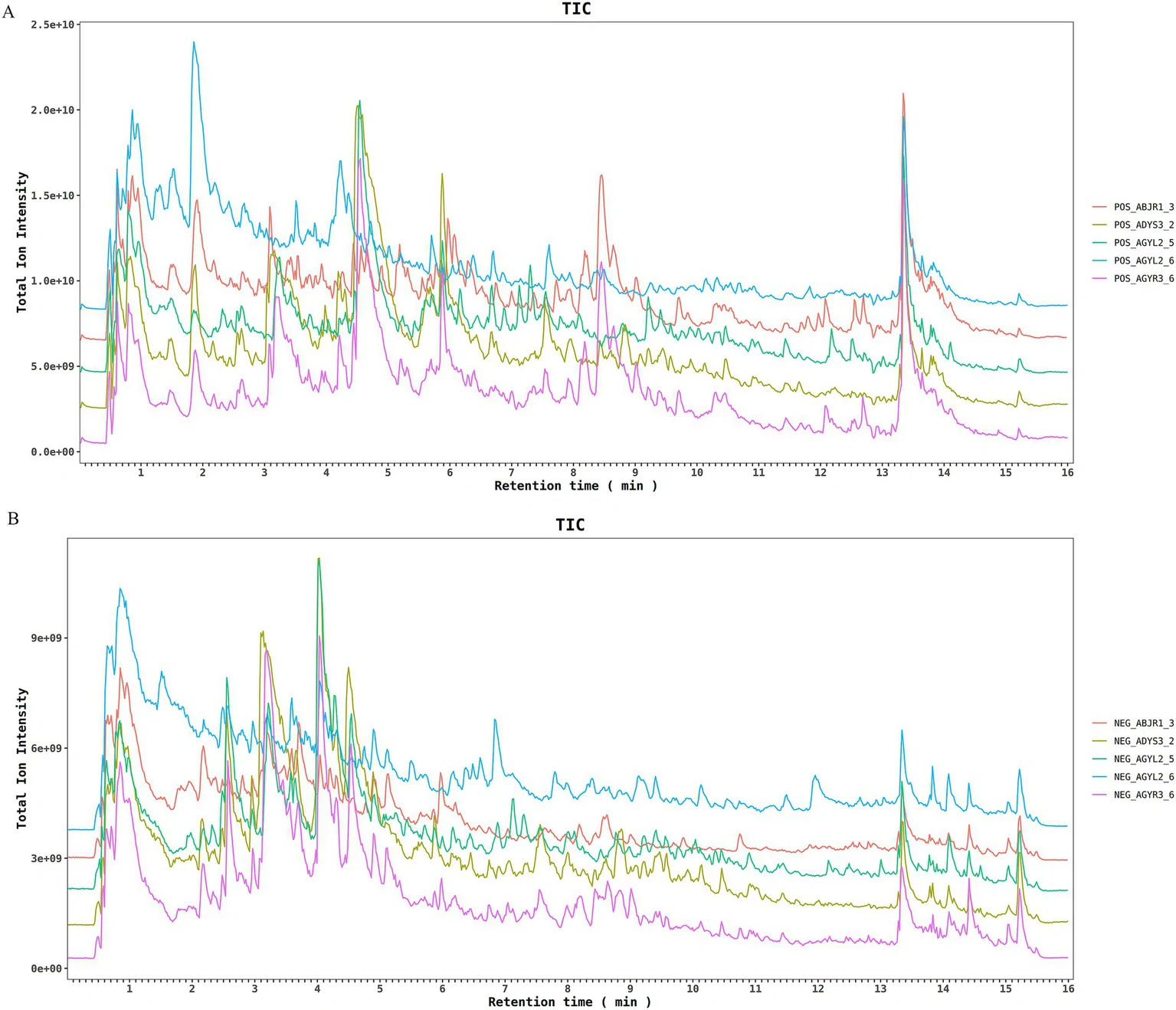
Total ion current (TIC) chromatograms of fermentation extracts from five highly active strains. **(A)** Positive ion mode. **(B)** Negative ion mode.

These identified metabolites were further classified into 16 categories, including 247 terpenoids, 53 flavonoids, 126 phenolic acids and their derivatives, 139 lipids, 31 carbohydrates and their derivatives, 44 amino acids and their derivatives, 79 organic acids and their derivatives, 37 coumarins and their derivatives, 5 lignans and their derivatives, 22 indoles and their derivatives, 12 nucleotides and their derivatives, 29 steroids and their derivatives, 17 quinones, 6 vitamins, 39 alkaloids and their derivatives, and 184 other compounds (Figure 7A).

**Figure 7:**
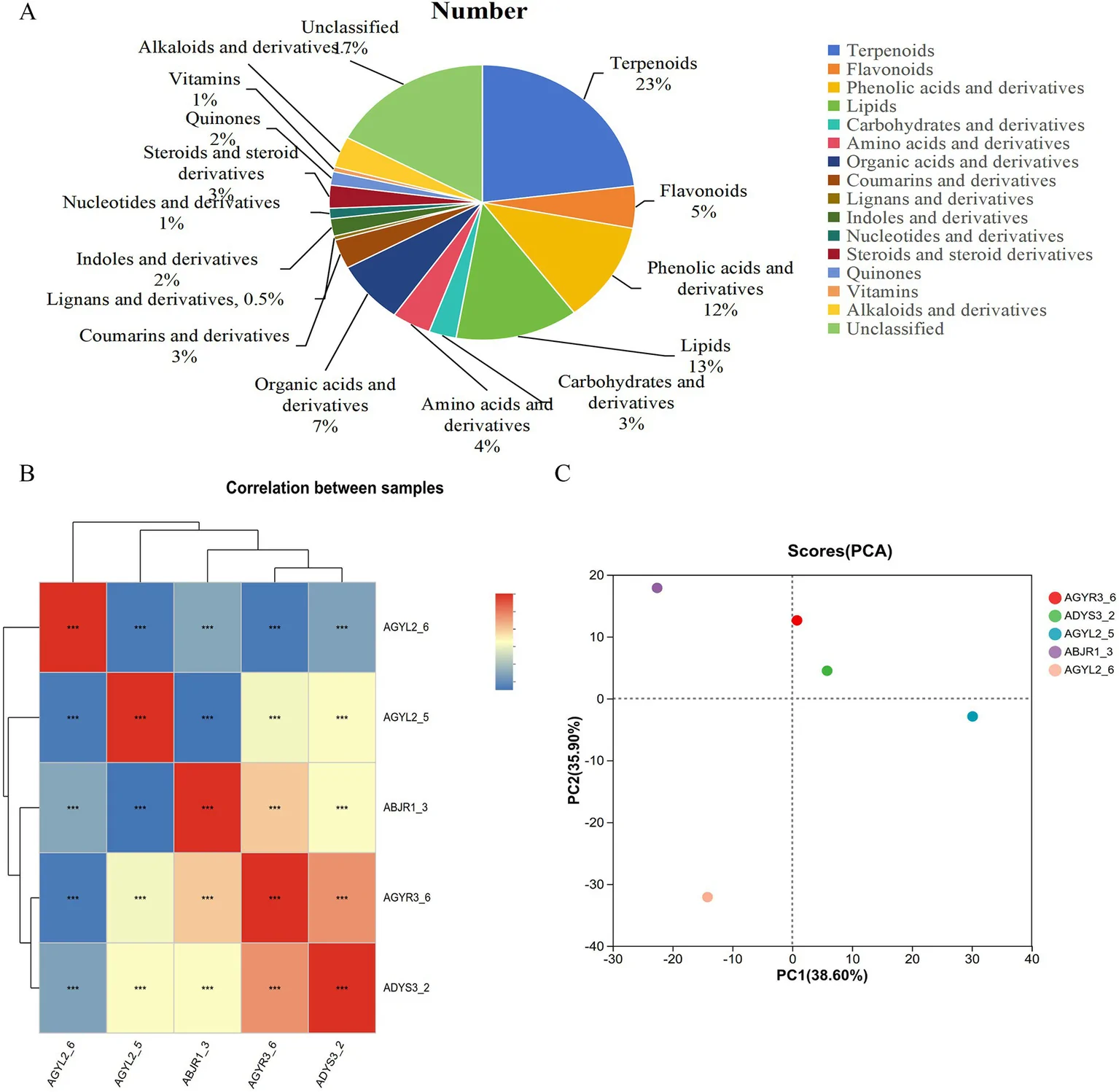
**(A)** Distribution of secondary metabolite classes. **(B)** Pearson correlation heatmap of metabolomes of the five strains. **(C)** Principal component analysis (PCA) score plot.

To evaluate the similarities and differences among the metabolomes of the five strains, Pearson correlation analysis was performed (Figure 7B). AGYL2-6 exhibited a low correlation with the other four strains, and its metabolite profile was significantly distinct from the others, which may explain its strong α-glucosidase inhibitory activity. AGYR3-6 and ADYS3-2 showed a high correlation, and both displayed strong antioxidant activity, suggesting that they may contain phenolic acid or flavonoid components. Principal component analysis (PCA) revealed that PC1 and PC2 explained 38.60 and 35.90% of the total variance, respectively, effectively discriminating the five strains (Figure 7C). AGYL2-6 deviated significantly from the other strains along PC1, forming an independent cluster. AGYR3-6 and ADYS3-2 were adjacent in the PC1/PC2 space, indicating a high similarity in their metabolite composition.

### 3.9 Chemical composition and structural analysis

#### 3.9.1 Identification of terpenoid metabolites

A total of 247 terpenoid metabolites were identified from the five strains, among which Secoxyloganin ranked highly in terms of response intensity and match score (Figure 8A). It was therefore selected as a representative to illustrate the possible fragmentation pathways of terpenoid metabolites. Under negative ion mode, the deprotonated molecular ion was observed at [M + H]- m/z 404.13, corresponding to the molecular formula C_17_H_24_O_11_. The fragmentation of this compound may proceed via two pathways: the parent ion undergoes cleavage of the glycosidic bond with the loss of a glucose moiety, generating the fragment ion at m/z 242.08; the resulting secoiridoid aglycone then undergoes skeletal rearrangement and decarboxylation to produce the base peak characteristic ions at m/z 162.08 and m/z 119.07, which can further lose H₂O to yield m/z 101.06; the cleaved glucose residue undergoes successive ring-opening and decarbonylation reactions, gradually generating a series of small oxygen-containing fragment ions, such as m/z 89.02 and 59.01.

**Figure 8:**
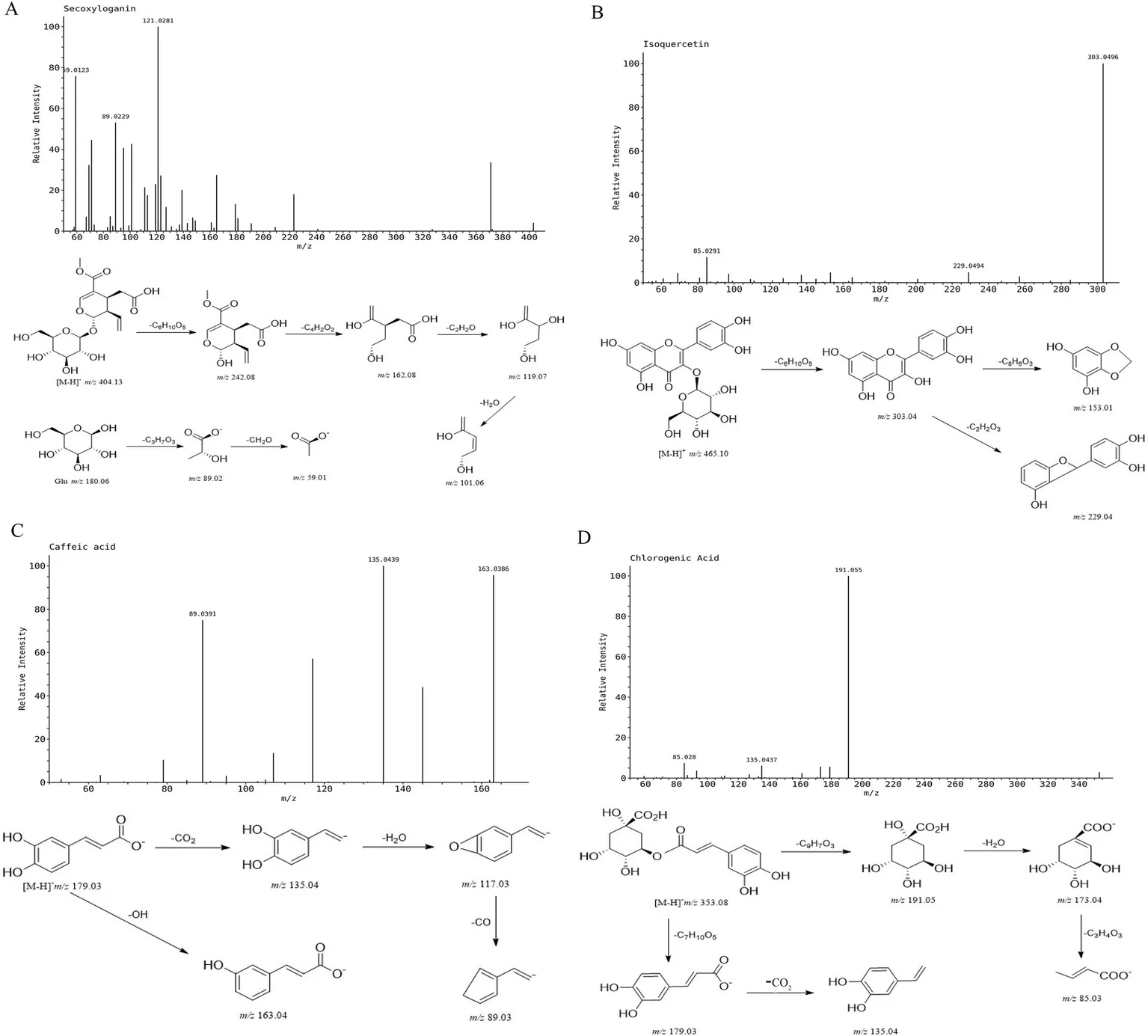
The degradation pathways. Secoxyloganin **(A)**, Isoquercetin **(B)**, Caffeic acid **(C)**, and Chlorogenic acid **(D)**.

#### 3.9.2 Identification of flavonoid metabolites

In this study, flavonoids were selected as the representative characteristic metabolites commonly present in the five strains, with isoquercetin chosen as an example to elucidate the fragmentation pathway of flavonoid compounds (Figure 8B). In positive ion mode, the protonated molecular ion [M + H]^+^ was observed at m/z 465.10, corresponding to the molecular formula C_21_H_20_O_12_. The fragmentation of this compound primarily follows one pathway: the parent ion first undergoes cleavage of the glucosidic bond, losing one glucose moiety to yield the quercetin aglycone, which appears as the base peak at m/z 303.0496. Subsequently, the aglycone undergoes RDA fragmentation, generating the characteristic flavonoid fragment ions at m/z 153.0181 and 229.0494.

#### 3.9.3 Identification of phenolic acid metabolites

Phenolic acids were identified as the characteristic metabolic components shared among the five strains, among which caffeic acid and chlorogenic acid received relatively high match scores. Therefore, these two compounds were selected as representatives to illustrate the possible fragmentation pathways of phenolic acid metabolites.

Under positive ion mode, the protonated molecular ion of caffeic acid was observed at [M + H] + m/z 179.03, with the molecular formula C_9_H_8_O_4_ (Figure 8C). Its fragmentation may proceed via two pathways: the parent ion loses one OH to generate the fragment ion at m/z 163.04; alternatively, the parent ion first loses one molecule of CO_2_ to yield m/z 135.04, which subsequently undergoes the loss of H_2_O to form m/z 117.03, and finally, after cleavage of the benzene ring side-chain skeleton with the loss of CO, the fragment ion at m/z 89.03 is produced.

Under negative ion mode, the deprotonated molecular ion of chlorogenic acid was observed at [M-H]- m/z 353.08, with the molecular formula C_16_H_18_O_9_ (Figure 8D). Two possible fragmentation pathways were proposed: the parent ion cleaves the ester bond between the caffeoyl group and quinic acid, losing the caffeoyl moiety to generate the quinic acid fragment ion at m/z 191.05; this fragment subsequently undergoes dehydration and ring cleavage to yield the characteristic ions at m/z 173.04 and 85.03; alternatively, the parent ion preferentially undergoes cleavage of the caffeoyl ester bond to generate the caffeic acid ion at m/z 179.03, which further loses CO_2_ to form the characteristic ion at m/z 135.04.

## 4 Discussion

This study isolated 703 culturable endophytic fungi from *Ardisia crenata* Sims, spanning 3 phyla and 96 genera, demonstrating substantial species diversity. Among these, Ascomycota predominated with an absolute advantage of 91.0%, a finding consistent with most studies on endophytic fungi in medicinal plants (Liu et al., 2025; An et al., 2020). At the genus level, *Diaporthe*, *Colletotrichum*, and *Cladosporium* were identified as the dominant genera, collectively accounting for 52.07% of all isolated strains. This dominant taxa pattern aligns closely with the endophytic fungal community characteristics of *A. crenata* Sims revealed by high-throughput sequencing (Liu et al., 2025), suggesting that these genera likely represent the “core endophytic fungi” of *A. crenata Sims*, having formed long-term and stable symbiotic relationships with the host plant. Notably, *Diaporthe* and *Colletotrichum*, widely reported as dominant endophytic fungal genera in medicinal plants (An et al., 2020; Yehia, 2023), have been confirmed to possess the ability to produce bioactive metabolites similar to those of their host plants. This provides important taxonomic guidance for the subsequent screening of highly active strains from endophytic fungi of *A. crenata* Sims.

Geographical origin and tissue type are two key factors driving the structural differentiation of the endophytic fungal community in *A. crenata* Sims. In terms of geographical origin, the GY site yielded the highest number of isolated strains and exhibited the greatest diversity at the genus level, whereas the GX and GD sites showed relatively lower diversity and fewer unique genera. Alpha diversity analysis further supports this trend: the Shannon-Wiener index and Simpson index for GY and CS were significantly higher than those for other regions, indicating that *A. crenata* Sims in Guizhou harbors endophytic fungi with greater genetic variation potential and microenvironmental adaptability. PCA and similarity coefficient analyses revealed that BJ and GD had the highest similarity in community structure, while CS and HN exhibited the lowest. This geographically driven community differentiation may be closely related to differences in ecological factors such as climatic conditions, soil physicochemical properties, and altitude across sampling sites (Liu et al., 2025; Nakamura et al., 2023).

Regarding tissue type, a pronounced tissue-specific colonization pattern was observed: leaves yielded the highest number of isolated strains, followed by stems, roots, and fruits. At the genus level, *Cladosporium* dominated in roots, *Diaporthe* was absolutely predominant in stems, *Colletotrichum* was the dominant genus in leaves, and *Penicillium* showed the highest relative isolation frequency in fruits. Similarly, investigations on endophytic fungi isolated from the medicinal plant *Dillenia indica* L. revealed that *Colletotrichum gloeosporioides* and *Cladosporium cladosporioides* were the dominant species, with the highest isolation frequency observed in leaves, which is highly consistent with the finding in this study that leaf tissues yielded the greatest number of isolated strains (Kumar and Prasher, 2022). This tissue-specific distribution may reflect differences in physical structure, chemical environment, and immune defense intensity among different tissues (Rodriguez et al., 2009). The high leaf colonization by *Colletotrichum*, as a putative “core endophyte,” implies a tight symbiosis with *A. crenata* Sims leaves, likely driven by its colonization strategy and microenvironmental adaptation. The predominance of *Diaporthe* in stems may correlate with higher lignin content and distinct nutrient dynamics. These tissue-specific patterns enhance our understanding of community assembly and inform targeted screening of tissue-derived strains for bioactive discovery.

In this study, 20 strains with low homology were identified through sequence alignment. Among them, AGYL2-1 (*Waltergamsia harwoodiae*) showed only 88.48% homology, AGZR4-1 (*Coprinopsis cinerea*) 89.53%, and ACSR2-1 (*Hypomontagnella monticulosa*) 90.79%. Based on the widely adopted ITS sequence similarity thresholds in fungal taxonomy, these strains are highly likely to represent potential novel species. This finding not only enriches the species pool of endophytic fungi associated with *A. crenata* Sims but also provides valuable microbial resources for the subsequent discovery of structurally novel active natural products. Novel species often harbor unique metabolic pathways and may produce structurally novel secondary metabolites with distinctive bioactivities, making them important sources for the discovery of drug lead compounds (Newman and Cragg, 2015).

Activity-guided screening serves as a bridge connecting microbial resources with industrial applications. In this study, the antioxidant, uric acid-lowering, and hypoglycemic activities of endophytic fungi from *A. crenata* Sims were evaluated, leading to the successful identification of multiple bioactive candidate strains. Regarding antioxidant activity, strain AGYR3-6 (*Dictyocheirospora rotunda*) exhibited a DPPH radical scavenging rate of 96.64% ± 0.21% at 1.0 mg/mL, comparable to that of the positive control vitamin C. Meanwhile, strain ADYS3-2 (*Aspergillus aculeatus*) achieved an ABTS^+^ radical scavenging rate of 99.39% ± 1.00%. For uric acid-lowering activity, strain AGYL2-5 (*Muyocopron heveae*) showed a xanthine oxidase inhibition rate of 86.88% ± 0.35% at 2.0 mg/mL, indicating superior anti-hyperuricemia potential. With respect to hypoglycemic activity, strain ABJR1-3 (*Fusicolla filiformis*) exhibited an α-amylase inhibition rate of 84.63% ± 0.07%, while strain AGYL2-6 (*Mycoleptodiscus terrestris*) achieved an α-glucosidase inhibition rate as high as 97.31% ± 0.01%, significantly outperforming the positive control acarbose. In recent years, endophytic fungi have emerged as a promising source for discovering novel α-glucosidase inhibitors. Maehara et al. isolated (+)-eupenoxide and its 3-ketone derivatives from an endophytic *Aspergillus* sp. of *Catharanthus roseus*, which exhibited selective α-glucosidase inhibitory activity, corroborating that *Aspergillus* represents a key group capable of producing hypoglycemic active compounds (Maehara et al., 2026). Notably, the α-glucosidase inhibitory activity of AGYL2-6 ranks among the highest reported for endophytic fungi to date, demonstrating a clear advantage over *Nigrospora sphaerica* BRN 01 isolated from *Bauhinia purpurea* L. (Kantari et al., 2023). A study demonstrated that the genera *Penicillium*, *Aspergillus*, *Fusarium*, and *Trichoderma* are key endophytic fungal resources for discovering antioxidant and antidiabetic bioactive compounds. The high-activity strains screened in this study not only further enrich the fungal resources but also provide valuable candidates for industrial development. The α-glucosidase inhibitory activity of *Colletotrichum fructicola* endophyte in *Cinnamomum tamala* was enhanced by 1.27 times through response surface methodology, indicating that the *Colletotrichum* genus is also an important group for exploring hypoglycemic active components (Arora and Saxena, 2025). In this study, the *Colletotrichum* strain isolated from different tissues of *Ardisia crenata* Sims had the highest frequency, providing a clear strain resource direction for subsequent targeted screening of α-glucosidase inhibitors from this genus.

Non-targeted metabolomics analysis provided important clues for elucidating the chemical basis of the highly active strains. UPLC-MS/MS analysis of five highly active strains identified a total of 1,071 secondary metabolites, covering various compound classes including terpenoids, phenolic acids, flavonoids, coumarins, and others. Based on UPLC-ESI-MS/MS analysis, previous studies have reported that 822 endophytic fungal strains produced a total of 17,809 compounds, with approximately 61% showing order-level specificity, further corroborating the vast chemical diversity potential of endophytic fungi as a natural product repository (Maciá-Vicente et al., 2018). Furthermore, using UHPLC-HRMS/MS combined with molecular networking technology, it was found that hosts and endophytic fungi engage in complex interactions through specialized chemical signals, and metabolomic differences among distinct endophytic fungal strains reflect the functional differentiation of their ecological roles (Pellissier et al., 2023). Correlation analysis and PCA results showed that the metabolic profile of AGYL2-6 was distinctly different from those of the other four strains, which may underlie its α-glucosidase inhibitory activity. In contrast, AGYR3-6 and ADYS3-2 exhibited highly similar metabolomic profiles, and both demonstrated strong antioxidant activity, suggesting that they may be jointly enriched in phenolic acid or flavonoid antioxidant components. Molecular docking and ADMET analysis were conducted on 150 phenolic compounds derived from endophytic fungi. It was found that the phenolic compounds produced by endophytic fungi have significant potential as α-glucosidase inhibitors (Kannakazhi Kantari et al., 2024).

From an industrial application perspective, this study has multiple implications. The production of bioactive compounds through endophytic fungal fermentation offers advantages such as a short cycle, controllable conditions, ease of scale-up, and environmental friendliness. Using microbial fermentation as an alternative to traditional plant extraction not only helps avoid overexploitation of wild *A. crenata* Sims resources but also effectively addresses the bottleneck in raw material supply, providing valuable microbial resources for the in-depth discovery of novel bioactive natural products. Among the strains, AGYL2-6 exhibits α-glucosidase inhibitory activity comparable to that of acarbose, suggesting that it may contain α-glucosidase inhibitors and holds potential for development into diabetes therapeutic drugs or functional food additives. Nevertheless, this study still has certain limitations: current activity evaluations are primarily based on crude fermentation extracts, and the isolation, purification, and structural identification of active components have not yet been performed; thus, the specific chemical structures and mechanisms of action of the bioactive substances remain unclear. In addition, the fermentation process has not been optimized, and the actual yield needs further improvement.

## 5 Conclusion

This study employed the endophytic fungal community of *Ardisia crenata* Sims as a bioindicator to evaluate the plant’s industrial potential. A diverse assemblage of endophytic fungi (703 strains, 96 genera) was isolated, with *Diaporthe*, *Colletotrichum*, and *Cladosporium* identified as the dominant genera. Twenty strains exhibiting low ITS homology suggest that *A. crenata* Sims harbors previously undescribed fungal diversity. Bioactivity screening of fungal metabolites revealed potent antioxidant and enzyme inhibitory activities, including DPPH scavenging (96.64% by AGYR3-6), xanthine oxidase inhibition (86.88% by AGYL2-5), α-amylase inhibition (84.63% by ABJR1-3), and exceptional α-glucosidase inhibition (97.31% by AGYL2-6, outperforming acarbose). Metabolomic analysis confirmed distinct chemical profiles underlying these activities. Collectively, these findings provide strong evidence that *A. crenata* Sims possesses the metabolic capacity to support the production of high-value bioactive compounds, supporting its development as a sustainable industrial crop. Future work should focus on establishing optimized cultivation practices for *A. crenata* Sims and developing scalable extraction or fermentation processes for industrial production.

## Data Availability

The datasets presented in this study can be found in online repositories. The names of the repository/repositories and accession number(s) can be found in the article/Supplementary material.
